# Severity of Psychologic Stressors Reflects Course of Crohn’s Disease in Two Siblings

**DOI:** 10.7759/cureus.16533

**Published:** 2021-07-21

**Authors:** Jessica Budiselic, Mary Sadlek, Keon Simpson

**Affiliations:** 1 Clinical Sciences, Saint James School of Medicine, The Quarter, AIA; 2 Family Medicine, Community Regional Medical Center, Fresno, USA

**Keywords:** inflammatory bowel disease, crohn’s disease (cd), biochemistry molecular biology clinical biochemistry cancer research oxidative stress, ulcerative colıtıs

## Abstract

A significant rise in Crohn’s disease (CD) cases amongst first-degree relatives strongly suggests that inflammatory bowel disease (IBD) has a genetic component. Adherence to medical management is at the forefront for preventing disease relapse. However, given the role that stress places on the immune system, it is imperative to implore an individual’s psychologic stressors to prevent future disease complications. This case of CD in two siblings, male and female, highlights the unique stress experienced by each patient at the time of symptom onset, the severity of their inflammatory symptoms, and their course of disease over several years. The male patient suffered from gender dysphoria and social anxiety for over a decade and had more chronic stress and severe complications of his disease. On the other hand, the stressors faced by his sister were periodic in nature and symptoms resided once stressful periods ended. For select patients, it is indicative that referral for psychotherapy should be considered as an ongoing mainstay of management. This case is intended to highlight the need for including psychotherapy in addition to medical management in order to treat IBD holistically.

## Introduction

Crohn’s disease (CD) is a subtype of inflammatory bowel disease (IBD) which presents as bowel inflammation anywhere along the gastrointestinal tract as a result of an immune-mediated reaction. The inflammatory process typically involves one of the two patterns of disease: a fibrostenotic obstructing pattern or a penetrating fistulous pattern, both with varying degrees of management. It classically presents with non-caseating granulomas which largely differentiates this condition from ulcerative colitis (UC) which favors the rectum.

Research has suggested that the etiology of IBD has a multifactorial inheritance. A significant rise in familial CD highly dictates that IBD has a genetic component [1]. Anticipation has also been expressed in several cases of familial CD with the second affected generation acquiring their disease at an earlier time in life [2]. Additionally, imprinting has been demonstrated to be associated with CD [3-4] with a higher female-to-female transmission compared with female-to-male transmission rate [2].

The case presented highlights the disease course of IBD in two siblings -- male (younger) and female (older). This is the first case of IBD in both parents’ families and it is unique because the amount of stress experienced by each patient at the time of symptom onset correlates with the severity of IBD symptoms and their disease course. Of note, the female patient was undiagnosed for a longer period than her brother, but he experienced his symptoms at an earlier age. This article was previously presented as a poster presentation at the 2020 AAFP FMX Virtual event on October 13, 2020.

## Case presentation

An 18-year-old biracial male (African and Caucasian) presents to the emergency department (ED) for severe abdominal pain, cramping, and bright red blood-tinged diarrhea for the past three weeks. The patient has had >10 bowel movements per day consisting of loose stool containing blood and mucus. He reports the pain as a burning sensation most prominent in the lower right quadrant. The pain is worse with defecation and is not related to eating.

Significant medical history includes wisdom teeth removal four weeks prior with general anesthesia, atopic dermatitis, and allergic rhinitis. He is allergic to dust, pollen, and penicillin. Additionally, the patient suffers from gender dysphoria and social anxiety since recently moving away for university. The patient is a non-smoker, does not drink alcohol, or take illicit drugs. He is up to date with his immunizations, takes no medications, and his family history is non-contributory. Vitals are normal and a physical exam is noteworthy for a diffusely tender abdomen in the lower quadrants bilaterally. A colonoscopy with biopsy is performed revealing inflammation of the terminal ileum and part of the ascending colon determined to be CD. Findings were more severe than his sibling in nature.

Medical management began with budesonide 9 mg oral daily in preparation for adalimumab. The patient was on adalimumab 40 mg subcutaneous (SC) every four weeks for over two years until he was hospitalized for a right colonic abscess. The patient was effectively treated in the hospital for six days and then switched to ustekinumab 260 mg SC every four weeks over the course of one year with the persistence of symptoms. The patient was then given vendolizumab 300 mg IV every four weeks for five months which was non-therapeutic and later replaced with infliximab 400 mg IV every four weeks. The patient is currently on infliximab which has been most efficient in managing symptoms.

The patient’s older sister is 20 years old when she presents to the clinic for episodic abdominal pain. She reports the pain as stabbing and localizes it to the left lower quadrant one inch from the umbilicus. She has four to five bowel movements per day which are loose, non-bloody, and contain mucus. She denies fever, vomiting, or constipation. The patient is up to date with immunizations, has no significant past medical history, and takes no medications. The patient drinks alcohol, smokes cigarettes socially, and does not take illicit drugs. Vital signs are normal and a physical exam illustrates a tender abdomen in the epigastric and left lower quadrant. Initial labs including comprehensive metabolic panel, complete blood count, and urinalysis were insignificant. Further testing includes Helicobacter pylori blood test, digital rectal exam, and stool guaiac test which are negative. 

**Figure 1:**
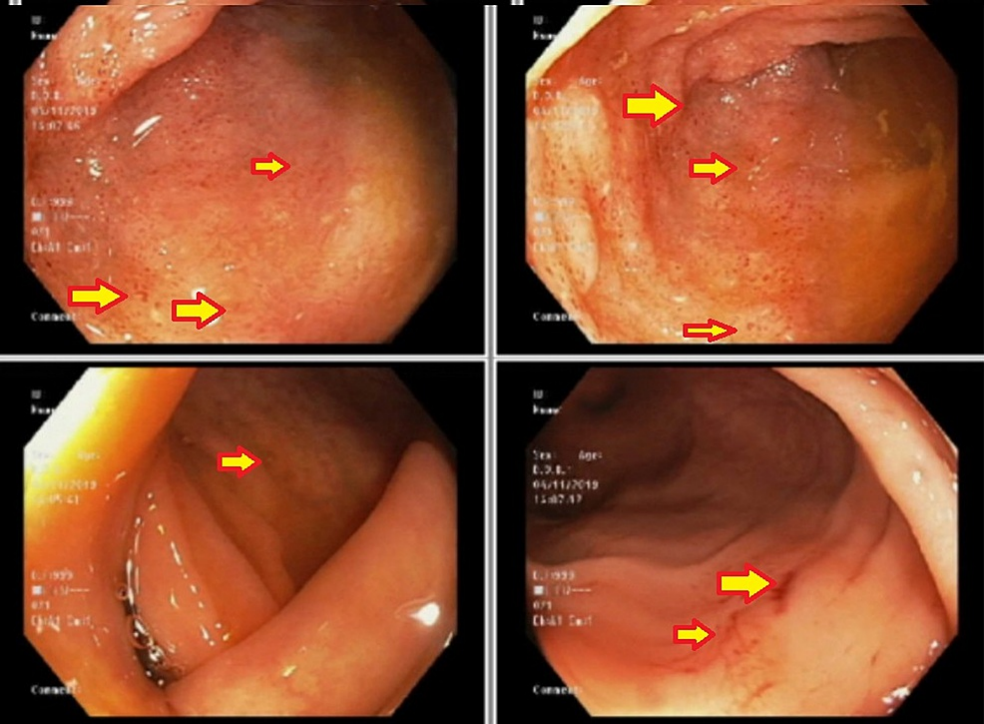
Terminal ileum with multiple petechial hemorrhages (arrows).

The patient is a full-time university student exposed to significant stress when the onset of symptoms occurred which resolved after cessation of the stressful period. Five years later under significant stress again, the same symptoms arise and a colonoscopy performed is positive for chronic inflammation of the terminal ileum. She takes mesalamine 1000 mg by mouth twice daily and reports no abdominal pain. Colonoscopy findings two years post-diagnosis reveal terminal ileum with active disease and a simple endoscopic score (SES) of five with no stricture of the colon and rectum (Figure 1). During this time, fecal calprotectin level measured is 67 µg/g and C-reactive protein (CRP) is 1 mg/L. 

## Discussion

The function of the stress response is to maintain homeostasis which consists of both physiological and behavioral adaptations. Increasing evidence has suggested that the hypothalamic pituitary adrenal (HPA) axis, autonomic nervous system (ANS), and enteric nervous system (ENS) interact directly with the immune system [5]. Psychological stress has long been reported to increase disease activity in IBD, and a study performed by Mawdsley and Rampton suggests that chronic stress, adverse life events, and depression increase the likelihood of relapse in patients with dormant IBD [6]. 

Acute stimulation of the sympathetic nervous system results in an almost instantaneous increase in both epinephrine and norepinephrine [7]. This response is followed by a rise in cortisol but both changes are maintained over only a few hours. Chronic sustained stress, such as those due to adverse life events, results in a prolonged increase in cortisol over several days which is characteristically associated with immunosuppression [8-11]. Both increased sympathetic tone and chronic stress have been associated with increased levels of CRP and erythrocyte sedimentation rate (ESR) signifying active inflammation [11-12].

The male patient had significant stressors for a longer duration than his sister and a more prolonged disease course. Six years after diagnosis, the male patient underwent gender reassignment surgery (male to female, MTF). During this time, the male patient commenced psychotherapy which remains ongoing weekly, and self-reports therapy as beneficial in managing stress. In parallel, the female patient presented with symptoms correlating with stressful periods and subsided after the stress period. Current symptoms continue to be mild.

There are several studies that have evaluated the clinical significance of psychodynamic and behavioral therapy on IBD [13-19]. The general consensus from these studies is that there are no statistically significant correlations between psychotherapy and the reduction of IBD symptoms. Of note, one limitation to several of these studies is that most of the therapy sessions were conducted in a group setting [15, 17-19]. This can result in the potential to miss individual stressors and influences psychotherapy may have on IBD.

## Conclusions

There are numerous factors to consider regarding why both patients had different courses of their disease which would require further testing, but it is difficult to ignore the role stress played. This case demonstrated that stressful life events coincided with the onset and relapse of symptoms for both patients. To highlight, the male patient had chronic stress for several years and had resistance to several medications. This is in contrast to his sister who had less significant, yet episodic stress responding well to mono-therapy. 

More research is required to study the individual effects of psychotherapy on a one-to-one basis which may provide benefit to some patients. For certain patients, early psychotherapy may have a positive impact on the effects of reducing stress and as a result inflammation in IBD. Further consideration for the psychological aspects of IBD should be explored to predict future disease complications. 

The relevance of this case report is to reiterate how crucial it is to discuss stressors with patients during each visit. This can be advantageous for potentially referring patients to the appropriate resources for stress management. There is benefit in creating a positive dialog between physicians and patients with IBD with the hope for patients to seek help during stressful periods in order to eliminate the risk of symptom exacerbation and improve overall well-being.
